# Adjoint Reactive GUI Programming

**DOI:** 10.1007/978-3-030-71995-1_15

**Published:** 2021-03-23

**Authors:** Christian Uldal Graulund, Dmitrij Szamozvancev, Neel Krishnaswami

**Affiliations:** 8 University of Oxford, Oxford, UK; 9 Sorbonne Université - LIP6, Paris, France; 10 IT University of Copenhagen, 2300 Copenhagen, Denmark; 11 University of Cambridge, Cambridge, CB3 0FD UK

**Keywords:** Linear Types, FRP, Asynchrony, GUIs

## Abstract

Most interaction with a computer is via graphical user interfaces. These are traditionally implemented imperatively, using shared mutable state and callbacks. This is efficient, but is also difficult to reason about and error prone. Functional Reactive Programming (FRP) provides an elegant alternative which allows GUIs to be designed in a declarative fashion. However, most FRP languages are synchronous and continually check for new data. This means that an FRP-style GUI will “wake up” on each program cycle. This is problematic for applications like text editors and browsers, where often nothing happens for extended periods of time, and we want the implementation to sleep until new data arrives. In this paper, we present an *asynchronous* FRP language for designing GUIs called $\lambda _{\mathsf {Widget}}$. Our language provides a novel semantics for widgets, the building block of GUIs, which offers both a natural Curry–Howard logical interpretation and an efficient implementation strategy.

## Introduction

Many programs, like compilers, can be thought of as functions – they take a single input (a source file) and then produce an output (such as a type error message). Other programs, like embedded controllers, video games, and integrated development environments (IDEs), engage in a dialogue with their environment: they receive an input, produce an output, and then wait for a new input that depends on the prior input, and produce a new output which is in turn potentially based on the whole history of prior inputs.

The usual techniques for programming interactive applications are often confusing, since different parts of the program are not written to interact via structured control flow (e.g., by passing and return values from functions). Instead, they communicate indirectly, via state-manipulating callbacks which are implicitly invoked by an event loop. This makes program reasoning very challenging, since each of aliased mutable state, higher-order functions, and concurrency is tricky on its own, and interactive programs rely upon their *combination*.

This challenge has led to a great deal of work on better abstractions for programming reactive systems. Two of the main lines of work on this problem are *synchronous dataflow* and *functional reactive programming*. The synchronous dataflow languages, like Esterel [5], Lustre [9], and Lucid Synchrone [28], feature a programming model inspired by Kahn networks. Programs are networks of stream-processing nodes which communicate with each other, each node consuming and producing a fixed number of primitive values at each clock tick. The first-order nature of these languages makes them strongly analysable, which lets them offer powerful guarantees on space and time usage. This means they see substantial use in embedded and safety-critical contexts.

Functional reactive programming, introduced by Elliott and Hudak [13], also uses time-indexed values, dubbed signals, rather than mutable state as its basic primitive. However, FRP differs from synchronous dataflow by sacrificing static analysability in favour of a much richer programming model. Signals are true first-class values, and can be used freely, including in higher-order functions and signal-valued signals. This permits writing programs with a dynamically-varying dataflow network, which simplifies writing programs (such as GUIs) in which the available signals can change as the program executes. Over the past decade, a long line of work has refined FRP via the Curry–Howard correspondence [21,18,17,19,20,10,1]. This approach views functional reactive programs as the programming counterpart for proofs of formulas in linear temporal logic [27], and has enabled the design of calculi which can rule out spacetime leaks [20] or can enforce temporal safety and liveness properties [10].

However, both synchronous dataflow and FRP (in both original and modal flavours) have a *synchronous* (or "pull") model of time – time passes in ticks, and the program wakes up on every tick to do a little bit more computation. This is suitable for applications in which something new happens at every time step (e.g., video games), but many GUI programs like text editors and spreadsheets spend most of their time doing nothing. That is, even at each event, most of the program will continue doing nothing, and we only want to wake up a component when an event directly relevant to it occurs. This is important both from a performance point of view, as well as for saving energy (and extending battery life). Because of this need, most GUI programs continue to be written in the traditional callbacks-on-mutable-state style.

In this paper, we give a reactive programming language whose type system both has a very straightforward logical reading, and which can give natural types to stateful widgets and the event-based programming model they encourage. We also derive a denotational semantics of the language, by first working out a semantics of widgets in terms of the operations that can be performed upon them and the behaviour they should exhibit. Then, we find the categorical setting in which the widget semantics should live, and by studying the structure this setup has, we are able to interpret all of the other types of the programming language.

***Contributions*** The contributions of this paper are:

– We give a descriptive semantics for widgets in GUI programming, and show that this semantics correctly models a variety of expected behaviours. For example, our semantics shows that a widget which is periodically re-set to the colour red is different from a widget that was only persistently set to the colour red at the first timestep. Our semantic model can show that as long as neither one is updated, they look the same, but that they differ if they are ever set to blue – the first will return to red at reset time, and the second will remain blue.

- From this semantics, we find a categorical model within which the widget semantics naturally fits. This model is a Kripke–Joyal presheaf semantics, which is morally a "proof-relevant" Kripke model of temporal logic.
- We give a concrete calculus for event-based reactive programming, which can be implemented in terms of the standard primitives for modern GUI programming, scene graphs (or DOM) which are updated via callbacks invoked upon events. We then show that our model can soundly interpret the types of our calculus in an entirely standard way, showing that the types of our reactive programming language can be interpreted as time-varying sets.
- Furthermore, this calculus has an entirely standard logical reading in terms of the Curry–Howard correspondence. It is a "linear temporal linear logic", with the linear part of the language corresponding to the Benton–Wadler [3] LNL calculus for linear logic, and the temporal part of the language corresponding to S4.3 linear temporal logic. We also give a proof term for the $S_t4.3$ axiom enforcing the linearity of time, and show that it corresponds to the `select` primitive of concurrent programming.

## The Language

We now present $\lambda_{\mathsf{Widget}}$ through the API of the $\mathsf{Widget}$ type. This API mirrors how one would work with a GUI at the browser level. An important feature of a well-designed GUI is that it should not do anything when not in use. In particular, it should not check for new inputs in each program cycle (*pull*-based reactive programming), but rather sleep until new data arrives (*push*-based reactive programming). Many FRP languages are *synchronous* languages and have some internal notion of a timestep. These languages are mostly pull-based, whereas more traditional imperative reactive languages are push-based. The former have clear semantics and are easy to reason about, the latter have efficient implementations. In $\lambda_{\mathsf{Widget}}$ we would like to combine these aspects and get a language that is easy to reason about with an efficient implementation.

In general, we think of a widget as a *state through time*, i.e., at each timestep, the widget is in some state which is presented to the user. The widget is modified by *commands*, which can update the state. To program with widgets, the programmer applies commands at various times.

The proper type system for a language of widgets should thus be a system with both state and time. If we consider what a *logic* for widgets should be, there are two obvious choices. A logic for state is linear logic [14], and a logic for time is linear temporal logic [27]. The combination of these two is the correct setting for a language of widgets, and, going through Curry–Howard, the corresponding type theory is a linear, linear temporal type theory.

***Widget API*** To work with widgets, we define a API which mirrors how one would work with a browser level GUI:

$$
\begin{aligned}
&\mathsf{newWidget} &&: \mathsf{I} \multimap \exists\,(i : \mathsf{Id}), \mathsf{Widget}\ i\\
&\mathsf{dropWidget} &&: \forall\,(i : \mathsf{Id}), \mathsf{Widget}\ i \multimap \mathsf{I}\\
&\mathsf{setColor} &&: \forall\,(i : \mathsf{Id}), \mathsf{F}\ \mathsf{Color} \otimes \mathsf{Widget}\ i \multimap \mathsf{Widget}\ i\\
&\mathsf{onClick} &&: \forall\,(i : \mathsf{Id}), \mathsf{Widget}\ i \multimap \mathsf{Widget}\ i \otimes \Diamond\,\mathsf{I}\\
&\mathsf{onKeypress} &&: \forall\,(i : \mathsf{Id}), \mathsf{Widget}\ i \multimap \mathsf{Widget}\ i \otimes \Diamond\,(\mathsf{F}\ \mathsf{Char})\\
&\mathsf{out} &&: \Diamond\,A \multimap \exists\,(n : \mathsf{Time}), A\,@\,n\\
&\mathsf{into} &&: \exists\,(n : \mathsf{Time}), A\,@\,n \multimap \Diamond\,A\\
&\mathsf{split} &&: \forall\,(i : \mathsf{Id})\ (t : \mathsf{Time}), \mathsf{Widget}\ i \multimap \mathsf{Prefix}\ i\ t \otimes (\mathsf{Widget}\ i)\,@\,t\\
&\mathsf{join} &&: \forall\,(i : \mathsf{Id})\ (t : \mathsf{Time}), \mathsf{Prefix}\ i\ t \otimes (\mathsf{Widget}\ i)\,@\,t \multimap \mathsf{Widget}\ i
\end{aligned}
$$

The first two commands creates and deletes widgets, respectively. The $\multimap$ should be understood as *state passing*. We read the type of newWidget as "consuming no state, produce a new identifier index and a widget with that identifier index". The identifier indices are used to ensure the correct behavior when using the split and join commands explained below. The existential quantification describes the *non-deterministic* creation of an identifier index. The use of non-determinism is crucial in our language and will be explaining in further detail in section 1. Since $\lambda_{\mathsf{Widget}}$ has a linear type system, we need an explicit construction to delete state. For widgets, this is dropWidget. The type is read as "for any identifier index, consume a widget with that identifier index and produce nothing".

The first command that modifies the state of a widget is setColor. Here we see the adjoint nature of the calculus with F Color. A color is itself *not* a linear thing, and as such, to use it in the linear setting, we apply F, which moves from the non-linear (Cartesian) fragment and into the linear fragment. The second new thing is the linear product $\otimes$. This differs from the regular non-linear product in that we do not have projection maps. Again, because of the linearity of our language, we cannot just discard state. We can now read the type of setColor as "Given a color and a identified widget, consume both and produce a new widget". The produced widget is the same as the consumed widget, but with the color attribute updated.

The next two commands, onClick and onKeypress, are roughly similar. Both register a handle on the widget, for a mouse click and a key press, respectively. Here we see the first use of the $\Diamond$ modality, which represents an *event*. The type $\Diamond A$ represents that *at some point in the future* we will receive something of type $A$. Importantly, because of the asynchronous nature of $\lambda_{\mathsf{Widget}}$, we do not know *when* it happens. We can then read the type of onClick as "Consuming an identified widget, produce an updated widget together with a mouse click event". The same holds for onKeypress except a key press event is produced.

The two commands out and into allows us to work with events in a more precise way. Given an event, we can use out to "unfold" it into an existential. The @ connective describes a type that is only available at a certain timestep, i.e., $A\,@\,n$ means "at the timestep $n$, a term of type $A$ will be available". The into commands is the reverse of out and turns an existential and an @ into an event.

Note the besides the above ways of constructing events, we can also turn any value into an event using the evt construction which is part of the core calculus. Given some element $a : A$, we get evt $a : \Diamond A$ which represents the event that returns immediately.

So far, we have only applied commands to a widget in the current timestep, but to program appropriately with widgets, we should be able to react to events and apply commands "in the future". This is exactly what the split and join commands allows us to do. The type of split is read as "Given any time step and any identified widget, split the widget into all the states *before* that time and the widget *at* that time". We denote the collection of states before a given time a *prefix* and give it the type Prefix. Given the state of the widget at a given timestep, we can now apply commands *at that timestep*. Note that both the prefix and the widget is indexed by the same identifier index. This is to ensure that when we use join, we combined the correct prefix and future.

***Widget Programming*** To see the API in action, we now proceed with several examples of widget programming. For each example, we will add a comment on each line with the type of variables, and then explain the example in text afterwards.

One of the simplest things we can do with a widget is to perform some action when the widget is clicked. In the following example, we register a handler for mouse clicks, and then we use the click event to change the color of the widget to red at the time of the click. To do this, we use the out map to get the time of the event, then we split the widget and apply setColor at that point in the future.

```
1  turnRedOnClick : ∀ (i : Id), Widget i ⊸ Widget i
2  turnRedOnClick i w0 =
3    let (w1, c0)          = onClick i w0 in      -- w1 : Widget i, c0 : ◇I
4    let unpack (x, c1)    = out c0 in            -- x : Time, c1 : I @ x
5    let c2 @ x            = c1 in                -- c2 : I at x
6    let ⟨⟩ @ x            = c2 in
7    let (p, w2)           = split i x w1 in      -- p : Prefix i x, w2 : Widget i @ x
8    let w3 @ x            = w2 in                -- w3 : Widget i at time x
9    let w4                =                      -- w4 : Widget i @ x
10       (setColor (F Red) w3) @ x in
11   join i x (p, w4)
```

To see why this type checks, we go through the example line by line. In line 3, we register a handle for a mouse click on the widget. In line 4, we turn the click event into an existential. In line 5, we get $c_2$ which is a binding that is only available at the timestep $x$. Since we only need the *time* of the click, we discharge the click itself in line 6. In line 7 and 8, we split the widget using the timestep $x$ and bind $w_3$ to the state of the widget at that timestep. In line 9-10, we change the color of the widget to red at $x$ and in line 11 we recompose the widget.

In general, we will allow pattern matching in eliminations and since widget identity indices can always be inferred, we will omit them. In this style, the above example become:

```
turnRedOnClick : ∀ (i : Id), Widget i ⊸ Widget i
turnRedOnClick w0 =
  let (w1, c0)            = onClick w1 in    -- w1 : Widget i, c0 : ◇I
  let unpack (x, ⟨⟩ @ x)  = out c0 in        -- x : Time
  let (p, w2 @ x)         = split x w1 in    -- p : Prefix i x, w2 : Widget i at time x
  join x (p, (setColor (F Red) w2) @ x)
```

We will use the same sugared style throughout the rest of the examples.

The above example turns a widget red exactly at the time of the mouse click, but will not do anything with successive clicks. To also handle further mouse clicks, we must register an event handler *recursively*. This is a simple modification of the previous code:

```
keepTurningRed : ∀ (i : Id), Widget i ⊸ Widget i
keepTurningRed w0 =
  let (w1, c0)            = onClick w1 in    -- w1 : Widget i, c0 : ◇I
  let unpack (x, ⟨⟩ @ x)  = out c0 in        -- x : Time
  let (p, w2 @ x)         = split x w1 in    -- p : Prefix i x, w2 : Widget i at time x
  join (p, (setColor (F Red) (keepTurningRed w2) @ x))
```

By calling itself recursively, this function will make sure a widget will always turn red on a mouse click.

To understand the difference between two above examples, consider the code $\mathit{turnBlueOnClick}(\mathit{keepTurningRed}\ w)$, where $w$ is some widget. On the first click, the widget will turn blue, on the second click it will turn red and on any subsequent click, it will keep turning red, i.e., stay red unless further modified.

When working with widgets, we will often register multiple handlers on a single widget. For example, a widget should have one behavior for a click and another behavior for a key press. To choose between two events, we use the select construction. This construction is central to our language and how to think about a push-based reactive language.

Given two events, $t_1 : \Diamond A, t_2 : \Diamond B$, there are three possible behaviors: Either $t_1$ returns first, and we wait for $t_2$ or $t_2$ returns first and we wait for $t_1$ or they return at the same time. In general, we want to select between $n$ events, but if we need to handle all possible cases, this will give $2^n$ cases, so to keep the syntax linear in size, we will omit the last case. In the case events *actually* return at the same time, we do a non-deterministic choice between them. The syntax for select is

$$\mathsf{select}\ (t_1\ \mathsf{as}\ x \mapsto t_1' \mid t_2\ \mathsf{as}\ y \mapsto t_2')$$

where $x : A, y : B, t_1' : A \multimap \Diamond B \multimap \Diamond C$ and $t_2' : B \multimap \Diamond A \multimap \Diamond C$. The second important thing to understand when working with select is that given we are working with events, we do not actually know at which timestep the events will trigger, and hence, we do not know what the (linear) context contains. Thus, when using select, we will *only* know either $a : A, t_2 : \Diamond B$ or $t_1 : \Diamond A, b : B$. We can think of the select rule a *case-expression* that must respect time.

In the following example, we register two handlers, one for clicks and one for key presses, and change the color of the widget based on which returns first. We will only annotate the new parts.

```
1  widgetSelect : ∀ (i : Id), Widget i ⊸ Widget i
2  widgetSelect w0 =
3    let (w1, c)          = onClick w0 in        -- c : ◇I.
4    let (w2, k)          = onKeypress w1 in     -- k : ◇(F char).
5    let col              =                      -- col : ◇(F Color)
6      select
7        ( c as x → let ⟨⟩ = x in               -- x : I, k : ◇(F Color).
8                   let unpack (t, ⟨⟩ @ t)
9                      = out (mapE (fun F (_) → ⟨⟩) k) in
10                  evt (F Red)
11       | k as y → let F k′ = y in             -- y : F char, c : ◇I
12                  let unpack (t, ⟨⟩ @ t) = c in
13                  evt (F Blue))
14   let unpack (x, col′ @ x) = out col in       -- col′ : F Color at time x.
15   let (p, w3 @ x)      = split x w2 in
16   join (p, (setColor col′ w3) @ x)
```

In line 3 and 4, we register the two handlers. In line 5-13, we use the select construction. In the first case, the click happens first and we return the color red. In the second case, the key press happens first and we return the color blue. In both cases, because of the linear nature of the language, we need to discharge the unit and char, respectively, and the event that does not return first. In line 14, we turn the color event into an existential. In line 15, we use the timestep of the color event to split the widget, and in line 16, we change the color of the widget at that time and recompose it.

To see how $\lambda_{\mathsf{Widget}}$ differs from more traditional synchronous FRP languages, we will examine how to encode a kind of streams. Since our language is *asynchronous*, the stream type must be encoded as

$$\mathsf{Str}\ A := \nu\alpha.\Diamond(A \otimes \alpha)$$

This asynchronous stream will *at some point in the future* give a head and a tail. We do not know when the first element of the stream will arrive, and after each element of the stream is produced, we will wait an indeterminate amount of time for the next element. The reason why the stream type in $\lambda_{\mathsf{Widget}}$ must be like this is essentially that we want a *push-based* language, i.e., we do not want to wake up and check for new data in each program cycle. Instead, the program should sleep until new data arrives.

To show the difference between the asynchronous stream and the more traditional synchronous stream, we will look at some examples. With a traditional stream, a standard operation is zipping two streams: that is, given $\mathsf{Str}\ A$ and $\mathsf{Str}\ B$, we can produce $\mathsf{Str}\ A \times B$, which should be the element-wise pairing of the two streams. It should be clear that this is not possible for our asynchronous streams. Given two streams, we can wait until the first stream produces an element, but the second stream may only produce an element after a long period of time. Hence, we would need to buffer the first element, which is not supported in general. Remember, when using select, we can not use any already defined linear variables, since we do not know if they will be available in the future.

Rather than zipping stream, we can instead do a kind of *interleaving* as shown below. We use fold and unfold to denote the folding and unfolding of the fixpoint.

```
interleave : Str A ⊸ Str B ⊸ Str (A ⊕ B)
interleave xs ys = fold (
  select
    ( unfold xs as xs′ →
        let (x, xs′′) = xs′ in     -- xs′ : A ⊗ Str A, x : A, xs′′ : Str A
        evt (inl x, interleave xs′′ ys)
    | unfold ys as ys′ →
        let (y, ys′′) = ys′ in     -- ys′ : B ⊗ Str B, y : B, ys′′ : Str A
        evt (inr y, interleave xs ys′′)))
```

Here, we use select to choose between which stream returns first, and then we let that element be the first element of the new stream.

On the other hand, some of the traditional FRP functions on streams can be translated. For instance, we can map of function over a stream, given that *it is available at each step in time*:

```
map : F (G (A ⊸ B)) ⊸ Str A ⊸ Str B
map f0 xs =
  let F f1              = f0 in   -- f1 : G(A ⊸ B)
  let (y, (x, xs′) @ y) =         -- y : Time, x : A, xs′ : ◇Str A at time y
    out (unfold xs) in
  fold (evt ((runG f1) x, map f0 xs′))
```

The type $\mathsf{F}(\mathsf{G}(A \multimap B))$ is read as a linear function with no free variables that can be used in a non-linear fashion, i.e., duplicated. This restriction to such "globally available functions" is reminiscent of the "box" modality in Bahr et al. [1] and Krishnaswami [20], and the F and G construction can be understood as decomposing the box modality into two separate steps. This relationship will be made precise in the logical interpretation of $\lambda_{\mathsf{Widget}}$ in section 1

As a final example, we will show how to dynamically update the GUI, i.e., how to add new widgets on the fly. Before we can give the example, we need to extend our widget API, to allow composition of widgets. To that end, we add the vAttach command to our API.

$$\mathsf{vAttach} : \forall(i, j : \mathsf{Id}), \mathsf{Widget}\ i \multimap \mathsf{Widget}\ j \multimap \mathsf{Widget}\ i$$

This command should be understood as an abstract version the div tag in HTML. In the following example, we think of the widget as a simple button that when clicked, will create a new button. When *any* of the buttons gets clicked, a new button gets attached.

```
1 buttonStack : ∀ i, Widget i ⊸ Widget i
2 buttonStack w0 =
3   let (w1, c)      = onClick w0 in
4   let (x, ⟨⟩ @ x)  = out e in
5   let (p, w2 @ x)  = split x w1 in
6   let w3           = (let (y, w) = newWidget ⟨⟩ in
7                        vAttach w2 (buttonStack w)) @ x in
8   join (p, w3)
```

The important step here is in line 6 and 7. Here the new button is attached at the time of the mouse click, and *buttonStack* is called recursively on the newly created button.

## Formal Calculus

This sections gives the rules, meta-theory and logical interpretation of $\lambda_{\mathsf{Widget}}$. Briefly, the language is a mixed linear-non-linear adjoint calculus in the style of Benton–Wadler [4,3]. The non-linear fragment, also called Cartesian in the following, is a minimal simply typed lambda calculus whereas the linear fragment contains several non-standard judgments used for widget programming.

***Contexts and Typing Judgments*** We have three typing judgments: one for indices, one for Cartesian (non-linear) terms, and one for linear terms. These are distinguished by a subscript on the turnstile, $i$ for indices, $c$ for Cartesian terms and $l$ for linear terms. These depend on different contexts. The index judgment depends only on a index context, whereas the Cartesian and linear judgments depends on both an index and a linear and/or a Cartesian context. The rules for context formation is given in Figure 1. These are mostly standard except for the dependence on a previously defined context and the fact that the linear context contains variables of the form $a :_\tau A$, i.e., temporal variables. The judgment $a :_\tau A$ is read as "$a$ has the type $A$ at the timestep $\tau$". In the linear setting we will write $a : A$ instead of $a :_0 A$, i.e., a judgment in the current timestep.

Indices: $$\frac{}{\vdash_i \cdot} \qquad \frac{\vdash_i \Theta \qquad s \notin \mathsf{dom}(\Theta) \qquad \sigma \in \{\mathsf{Id}, \mathsf{Time}\}}{\vdash_i \Theta, s : \sigma}$$

Cartesian: $$\frac{}{\cdot \vdash_c} \qquad \frac{\Theta \vdash_c \Gamma \qquad x \notin \mathsf{dom}(\Gamma) \qquad \Theta \vdash_c X}{\Theta \vdash_c \Gamma, x : X}$$

Linear: $$\frac{}{\cdot \vdash_l} \qquad \frac{\Theta \vdash_l \Delta \qquad x \notin \mathsf{dom}(\Delta) \qquad \Theta \vdash_l A \qquad \Theta \vdash_i \tau : \mathsf{Time}}{\Theta \vdash_l \Delta, a :_\tau A}$$

**Fig. 1.** Context Formation

The index judgment describes how to introduce indices. The typing rules are given in Figure 2. The judgment $\Theta \vdash_i \tau : \sigma$ contains a single context, $\Theta$, for index variables. There are only two sorts of indices, identifiers and timesteps.

**Index Judgments:**

$$\frac{\tau \in \mathsf{Time}}{\Theta \vdash_i \tau : \mathsf{Time}}\ \textsc{Time} \qquad \frac{\iota \in \mathsf{Id}}{\Theta \vdash_i \iota : \mathsf{Id}}\ \textsc{Id} \qquad \frac{i : \sigma \in \Theta}{\Theta \vdash_i i : \sigma}\ \textsc{Var}$$

**Fig. 2.** Index Typing rules

The Cartesian judgment describes the Cartesian, or non-linear, fragment. This is a minimal simply typed lambda calculus with the addition of the $\mathsf{G}$ type, used for moving between the linear and Cartesian fragment, and explained further below. The judgment $\Theta; \Gamma \vdash_c t : A$ has two contexts; $\Theta$ for indices and $\Gamma$ for Cartesian variables.

The linear fragment is most of the language, and a selection of typing rules is given in Figure 3. The judgment is done w.r.t three contexts, $\Theta$ for index variables, $\Gamma$ for Cartesian variables and $\Delta$ for linear variables. Many of the rules are standard for a linear calculus, except for the presence of the additional contexts. We will not describe the standard rules any further.

The first non-standard rule is for $\Diamond$. The introduction and elimination rules follow from the fact that $\Diamond$ is a non-strong monad. More interesting is the $\mathsf{select}$ rule. Here we see the formal rule corresponding to the informal explanation in section 1. The important thing here is that we can not use any previously defined linear variable when typing $t_1'$ and $t_2'$, since we do not actually know *when* the typing happens. Note, we can see the $\mathsf{select}$ rule as a binary version of the $\Diamond$ let-binding. This could be extended to an $n$-ary version, but we do not do this in our core calculus. The rules for $A \,@\, \tau$ shows how to move between the judgment $t : A \,@\, \tau$ and $t :_\tau A$. That is, moving from knowing in the current timestep that $t$ will have the type $A$ at time $\tau$ and knowing at time $\tau$ that $t$ has type $A$. The ($\mathsf{F}$ -I), ($\mathsf{F}$ -E), ($\mathsf{G}$ -I) and ($\mathsf{G}$ -E) rules show the adjoint structure of the language. The ($\mathsf{G}$ -I) rule takes a closed linear term of type $A$ and gives it the Cartesian type $\mathsf{G}\ A$. Note, because it has no free linear variables, it is safe to duplicate. The ($\mathsf{G}$ -E) rule lets us get an $A$ without needing any linear resources. Conversely, the ($\mathsf{F}$ -I) rule embeds a intuitionistic term into the linear fragment and the ($\mathsf{F}$ -E) rule binds an intuitionistic variable to let us freely use the value. The ($\mathsf{Delay}$) rule shows what happens when we actually *know* the timestep. The important part is $\Delta' = \Delta \downarrow^\tau$ which means two things. One, all the variables in $\Delta$ are on the form $a :_\tau A$, i.e., judgments at time $\tau$ and two, we shift $\Delta$ into the future such that all the variables of $\Delta'$ is of the form $a : A$. The way to understand this is, if all the variables in $\Delta$ are typed at time $\tau$ and the conclusion is at time $\tau$, it is enough to "move to" time $\tau$ and then type w.r.t that timestep. Finally, we have ($\mathsf{I}_\tau$-E) and ($\otimes_\tau$-E). These allow us to work with linear unit and products at time $\tau$. These are added explicitly since they can not be derived by the other rules, and are needed for typing certain kinds of programs.

***Unfolding Events to Exists*** The type system as given above contains both $\Diamond A$ and $A \,@\, k$, as two distinct ways to handle time. The former means that

$$\frac{\Theta \vdash_i \tau : \mathsf{Time} \qquad \Theta; \Gamma; \Delta \vdash_l t :_\tau A}{\Theta; \Gamma; \Delta \vdash_l t \mathbin{@} \tau : A \mathbin{@} \tau} \ (@\text{-I})$$

$$\frac{\Theta \vdash_i t : \mathsf{Time} \qquad \Theta; \Gamma; \Delta_1 \vdash_l t_1 : A \mathbin{@} \tau \qquad \Theta; \Gamma; \Delta_2, a :_\tau A \vdash_l t_2 : B}{\Theta; \Gamma; \Delta_1, \Delta_2 \vdash_l \mathsf{let}\ a \mathbin{@} \tau = t_1\ \mathsf{in}\ t_2 : B} \ (@\text{-E})$$

$$\frac{\Theta; \Gamma \vdash_c e : \mathsf{G}\ A}{\Theta; \Gamma; \cdot \vdash_l \mathsf{runG}\ e : A} \ (\mathsf{G}\text{-E}) \qquad \frac{\Theta; \Gamma \vdash_c e : X}{\Theta; \Gamma; \cdot \vdash_l \mathsf{F}\ e : \mathsf{F}\ x} \ (\mathsf{F}\text{-I})$$

$$\frac{\Theta; \Gamma; \Delta_1 \vdash_l t_1 : \mathsf{F}\ X \qquad \Theta; \Gamma, x : X; \Delta_2 \vdash_l t_2 : B}{\Theta; \Gamma; \Delta_1, \Delta_2 \vdash_l \mathsf{let}\ \mathsf{F}\ x = t_1\ \mathsf{in}\ t_2 : B} \ (\mathsf{F}\text{-E})$$

$$\frac{\Theta, i : \sigma; \Gamma; \Delta \vdash_l t : A}{\Theta; \Gamma; \Delta \vdash_l \Lambda(i : \sigma).t : \forall(i : \sigma).A} \ (\forall\text{-I}) \qquad \frac{\Theta \vdash_i s : \sigma \qquad \Theta; \Gamma; \Delta \vdash_l t : \forall(i : \sigma).A}{\Theta; \Gamma; \Delta \vdash_l t_s : \{s/i\}A} \ (\forall\text{-E})$$

$$\frac{\Theta \vdash_i s : \sigma \qquad \Theta; \Gamma; \Delta \vdash_l t : \{s/i\}A}{\Theta; \Gamma; \Delta \vdash_l \{s, t\} : \exists(i : \sigma).A} \ (\exists\text{-I})$$

$$\frac{\Theta; \Gamma; \Delta_1 \vdash_l t_1 : \exists(i : \sigma).A \qquad \Theta, s : \sigma; \Gamma; \Delta_2, a : \{s/i\}A \vdash_l t_2 : B}{\Theta; \Gamma; \Delta_1, \Delta_2 \vdash_l \mathsf{let\ unpack}\ \{s, a\} = t_1\ \mathsf{in}\ t_2 : B} \ (\exists\text{-E})$$

$$\frac{\begin{array}{c}\Theta; \Gamma; \Delta_1 \vdash_l t_1 : \Diamond A \qquad \Theta; \Gamma; \Delta_2 \vdash_l t_2 : \Diamond B \\ \Theta; \Gamma; a : A, t_2 : \Diamond B \vdash_l t_1' : \Diamond C \qquad \Theta; \Gamma; b : B, t_1 : \Diamond A \vdash_l t_2' : \Diamond C\end{array}}{\Theta; \Gamma; \Delta_1, \Delta_2 \vdash_l \mathsf{select}\ (t_1\ \mathsf{as}\ a \mapsto t_1' \mid t_2\ \mathsf{as}\ b \mapsto t_2') : \Diamond C} \ (\textsc{select})$$

$$\frac{\Theta \vdash_i \tau : \mathsf{Time} \qquad \Delta' = \Delta \downarrow^\tau \qquad \Theta; \Gamma; \Delta' \vdash_l t : A}{\Theta; \Gamma; \Delta \vdash_l t :_\tau A} \ (\textsc{delay})$$

$$\frac{\Theta \vdash_i \tau : \mathsf{Time} \qquad \Theta; \Gamma; \Delta_1 \vdash_l t_1 :_\tau \mathsf{I} \qquad \Theta; \Gamma; \Delta_2 \vdash_l t_2 : B}{\Theta; \Gamma; \Delta_1, \Delta_2 \vdash_l \mathsf{let}\ \langle\rangle \mathbin{@} \tau = t_1\ \mathsf{in}\ t_2 : B} \ (\mathsf{I}_\tau\text{-E})$$

$$\frac{\begin{array}{c}\Theta \vdash_i \tau : \mathsf{Time} \\ \Theta; \Gamma; \Delta_1 \vdash_l t_1 :_\tau A \otimes B \qquad \Theta; \Gamma; \Delta_2, a :_\tau A, b :_\tau B \vdash_l t_2 : C\end{array}}{\Theta; \Gamma; \Delta_1, \Delta_2 \vdash_l \mathsf{let}\ (a, b) \mathbin{@} \tau = t_1\ \mathsf{in}\ t_2 : C} \ (\otimes_\tau\text{-E})$$

**Fig. 3.** Selected Linear Typing rules something of type $A$ will arrive at *some* point in the future, whereas the latter means an $A$ arrives at a *specific* point in the future. The strength of $\diamond$ is that is gives easy and concise typing rules, whereas the strength of $A \mathbin{@} k$ is that it allows for a more precise usage of time. To connect these two, we add the linear isomorphism $\diamond A \cong \exists k. A \mathbin{@} k$ to our language, which is witnessed by out and into, as part of the widget API. This isomorphism is true semantically, but can not be derived in the type system. In particular, this isomorphism allows the select rule to be given with $\diamond$, while still allowing the use timesteps when working with the resulting event. If we were to give the equivalent definition using timesteps, one would need to have some sort of *constraint system* for deciding which events happens first. Avoiding such constraints also allows for a simpler implementation, as everything is our type system can be inferred.

***Meta-theory of Substitution*** The meta-theory of $\lambda_{\mathsf{Widget}}$ is given in the form of a series of substitution lemmas. Since we have three different contexts, we will end up with six different substitutions into terms. The Cartesian to Cartesian, Cartesian to linear and linear to linear are the usual notion of mutual recursive substitution. More interesting is the substitution of indices into Cartesian and linear terms and types. We prove the following lemma, showing that typing is preserved under index substitution:

**Lemma 1 (Preservation of Typing under Index Substitution).**

$$\frac{\zeta : \Theta' \to \Theta \qquad \Theta; \Gamma \vdash_c e : X}{\Theta'; \zeta(\Gamma) \vdash_c \zeta(e) : \zeta(X)} \qquad \frac{\zeta : \Theta' \to \Theta \qquad \Theta; \Gamma; \Delta \vdash_l t :_\tau A}{\Theta'; \zeta(\Gamma); \zeta(\Delta) \vdash_l \zeta(t) :_\tau \zeta(A)}$$

Both are these (and all other cases for substitution) are proved by a lengthy but standard induction over the typing tree. See the technical appendix for full proofs of all six substitution lemmas.

***Logical Interpretation*** Our language has a straightforward logical interpretation. The logic corresponding to the Cartesian fragment is a propositional intuitionistic logic, following the usual Curry–Howard interpretation. The logic corresponding to the substructural part of the language is a linear, linear temporal logic. The single-use condition on variables means that the syntax and typing rules correspond to the rules of intuitionistic linear logic (i.e., the first occurrence of linear in “linear, linear temporal”). However, we do not have a comonadic exponential modality $!A$ as a primitive. Instead, we follow the Benton–Wadler approach [4,3] and decompose the exponential into the composition of a pair of adjoint functors mediating between the Cartesian and linear logic.

In addition to the Benton–Wadler rules, we have a temporal modality $\diamond A$, which corresponds to the eventually modality of linear temporal logic (i.e., the second occurrence of “linear” in “linear, linear temporal logic”). This connective is usually written $F\,A$ in temporal logic, but that collides with the $\mathsf{F}$ modality of the Benton–Wadler calculus. Therefore we write it as $\diamond A$ to reflect its nature as a possibility modality (or monad). In our calculus, the axioms of S4.3 are derivable:

$$(T) : A \multimap \Diamond A$$
$$(4) : \Diamond\Diamond A \multimap \Diamond A$$
$$(.3) : \Diamond(A \otimes B) \multimap \Diamond((\Diamond A \otimes B) \oplus \Diamond(A \otimes \Diamond B) \oplus \Diamond(A \otimes B))$$

Since the ambient logic is linear, intuitionistic implication $X \to Y$ is replaced with the linear implication $A \multimap B$, and intuitionistic conjunction $X \wedge Y$ is replaced with the linear tensor product $A \otimes B$. It is easy to see that the first two axiom corresponds to the monadic structure of $\Diamond$, and the .3 axiom corresponds to the $\mathsf{select}$ rule (with our syntax for $\mathsf{select}$ corresponding to immediately waiting for and then pattern-matching on the sum type). In the literature, the .3 axiom is often written in terms of the box modality $\Box A$ [8], but we present it here in a (classically) equivalent formulation mentioning the eventually modality $\Diamond A$. We do not need to an explicit box modality $\Box A$, since the decomposition of the exponential $\mathsf{F}(\mathsf{G}A)$ from the linear-non-linear calculus serves that role.

In our system, *we do not offer* the next-step operator $\rhd A$. Since we model asynchronous programs, we do not let programmers write programs which wake up in a specified amount of time. We only offer an iterated version of this connective, $A @ n$, which can be interpreted as $\rhd^n A$, and our term syntax has no numeric constants which can be used to demand a specific delay.

Finally, the universal and existential quantifiers (in both the intuitionistic and linear fragments) are the usual quantifier rules for first-order logic.

## Semantics

In this section we give a denotational model for $\lambda_{\mathsf{Widget}}$. It is a linear-non-linear (LNL) hyperdoctrine [24,16] with the non-linear part being $\mathsf{Set}$ and the linear part being the category of internal relations over a suitable "reactive" category. The hyperdoctrine structure is used to interpret the quantification over indices. This model is nearly entirely standard: the most interesting thing is the reactive base category and the interpretation of widgets. It is well known that any symmetric monoidal closed category (SMCC) models multiplicative intuitionistic linear logic (MILL), and it is similarly well known that the category of relations over $\mathsf{Set}$ can be give the structure of a SMCC by using the Cartesian product as both the monoidal product and monoidal exponential. This construction lift directly to any category of internal relations over a category that is suitably "$\mathsf{Set}$-like", i.e., a topos. Our base category is a simple presheaf category, and hence, we use this construction to model the linear fragment of $\lambda_{\mathsf{Widget}}$.

***The Base Reactive Category*** The base reactive category is where the notion of time will arise and is it this notion that will be lifted all the way up to the LNL hyperdoctrine. The simplest model of "time" is $\mathsf{Set}^{\mathbb{N}}$, which can be understood as "sets through time" [23]. This can indeed by used as a model for a reactive setting, but for our purposes it is too simple, and further, depending on which ordering is considered for $\mathbb{N}$, may have undesirable properties for the reactive setting. Instead, we use the only slightly more complicated $\mathsf{Set}^{\mathbb{N}+1}$, henceforth denoted $\mathcal{R}$, where the ordering on $\mathbb{N}+1$ is the discrete ordering on $\mathbb{N}$ and 1 is related to everything else. Adding this "point at infinity" allows global reasoning about objects, an intuition that is further supported by the definition of the sub-object classifier below. Further, this model is known to be able to differentiate between least and greatest fixpoints [15], and even though we do not use this for $\lambda_{\mathsf{Widget}}$, we consider it a useful property for further work (see section 1). Objects in $\mathcal{R}$ can be visualized as

$$A = \quad \begin{array}{ccccc} & & A_\infty & & \\ & \swarrow^{\pi_1} & \downarrow^{\pi_2} & \searrow & \\ A_0 & & A_1 & & \cdots \end{array}$$

We can think of $A_\infty$ as the global view of the object and $A_n$ as the local view of the object at each timestep. Morphisms are natural transformations between such diagrams and the naturality condition means that having a map from $A_\infty$ to $B_\infty$ must also come with coherent maps at each timestep.

In $\mathcal{R}$ we define two endofunctors, which can be seen as describing the passage of time:

**Definition 1.** *We define the* later *and* previous *endofunctors on $\mathcal{R}$, denoted $\triangleright$ and $\triangleleft$, respectively:*

$$(\triangleright A)_n := \begin{cases} 1 & n = 0 \\ A_{n'} & n = n' + 1 \\ A_\infty & n = \infty \end{cases} \qquad\qquad (\triangleleft A)_n := \begin{cases} A_{n+1} & n \neq \infty \\ A_\infty & n = \infty \end{cases}$$

Note that when we apply the later functor, the global view does not change, but the local views are shifted forward in time.

**Theorem 1.** *The later and previous endofunctors form an adjunction.*

**Definition 2.** *The sub-object classifier, denoted $\Omega$, in $\mathcal{R}$ is the object*

$$\Omega_\infty = \mathcal{P}(\mathbb{N}) + 1 \qquad\qquad \Omega_n = \{0, 1\}$$

For each $n \in \mathbb{N}$, $\Omega_n$ denotes whether a given proposition is true at the $n$th timestep. $\Omega_\infty$ gives the "global truth" of a given proposition. The left injection is some subset of $\mathbb{N}$ that denotes at which points in time something is true. The right injection denotes that something is true "at the limit", and in particular, also at all timesteps. Note, a proposition can be true at all timesteps but not at the limit. This extra point at infinity is precisely what allows us the differentiate between least and greatest fixpoints.

***The Category of Internal Relations*** To interpret the linear fragment of the language, we will use the category of internal relations on $\mathcal{R}$. Given two objects $A$ and $B$ in $\mathcal{R}$, an *internal relation* is a sub-object of the product $A \times B$. This can equivalently by understood as a map $A \times B \to \Omega$. The category of internal relations in the category where the objects are the objects of $\mathcal{R}$ and the morphisms $A \to B$ are internal relations $A \times B \to \Omega$ in $\mathcal{R}$. We denote the category of internal relations as $\mathsf{Rel}_{\mathcal{R}}$.

**Theorem 2.** *Using* $A \otimes B = A \times B$ *and* $A \multimap B = A \times B$ *as monoidal product and exponential, respectively,* $\mathsf{Rel}_{\mathcal{R}}$ *is a symmetric monoidal closed category.*

**Theorem 3.** *There is an adjunction* $\triangleleft \vdash \triangleright$ *in* $\mathsf{Rel}_{\mathcal{R}}$ *where* $\triangleleft$ *and* $\triangleright$ *are the lifting of the previous and later functors from* $\mathcal{R}$ *to* $\mathsf{Rel}_{\mathcal{R}}$*.*

**Definition 3.** *We define the* iterated later modality *or the "at" connective as a successive application of the later modality.*

$$\triangleright^{0} A = A$$
$$\triangleright^{(k+1)} A = \triangleright(\triangleright^{k} A)$$

*and we will alternatively write* $A \,@\, k$ *to mean* $\triangleright^{k} A$*.*

**Definition 4.** *We define the* event *functor on* $\mathsf{Rel}_{\mathcal{R}}$ *as an iterated later.*

$$\Diamond A : \mathsf{Rel}_{\mathcal{R}} \to \mathsf{Rel}_{\mathcal{R}}$$
$$(\Diamond A)_{\infty} = A_{\infty}$$
$$(\Diamond A)_{n} = \Sigma(k : \mathbb{N}).(\triangleright^{k} A)_{n}$$

The event functor additionally carries a monadic structure (see [29] and the technical appendix).

**Theorem 4.** *We have the isomorphism* $\Diamond A \cong \Sigma(n : \mathbb{N}).A \,@\, n$ *for any* $A$

**Theorem 5.** *We have the following adjunctions between* $\mathsf{Set}$*,* $\mathcal{R}$ *and* $\mathsf{Rel}_{\mathcal{R}}$*:*

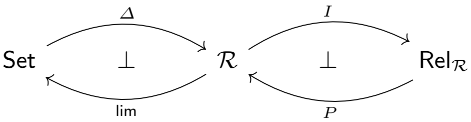

*where* $\Delta$ *is the constant functor,* $\mathsf{lim}$ *is the limit functor,* $I$ *is the inclusion functor and* $P$ *is the image functor. This induces an adjunction between* $\mathsf{Set}$ *and* $\mathsf{Rel}_{\mathcal{R}}$*.*

***The Widget Object*** One of the most important objects in $\mathsf{Rel}_{\mathcal{R}}$ is the *widget* object. This object is used to interpret widgets and prefixes. The widget object will be defined with respect to an ambient notion of identifiers, which we will denote $\mathsf{Id}$. These will be part of the hyperdoctrine structure define below, and for now, we will just assume such an object to exists. We will also use a notion of timesteps internal to the widget object. Note that this timestep is different from the abstract timestep used for defining $\mathsf{Rel}_{\mathcal{R}}$, but are related as defined below. We denote the abstract timesteps with $\mathsf{Time}$.

Before we can define the widget object, we need to define an appropriate object of commands. In our minimal Widget API, the only *semantic* commands will be $\mathsf{setColor}$, $\mathsf{onClick}$ and $\mathsf{onKeypress}$. The rest of the API is defined as morphisms on the widget object itself. To work with the semantics commands, we additionally need a *compatibility* relation. This relation describes what commands can be applied at the same time. In our setting this relation is minimal, but can in principle be used to encode whatever restrictions is needed for a given API.

**Definition 5.** *We define the command object as*

$$\mathsf{Cmd} = \{(\mathsf{setColor}, color), \mathsf{onClick}, \mathsf{onKeypress}\}$$

*where color is an element of a "color" object. The compatibility relations are:*

$$(op, arg) \bowtie (op', arg') \text{ iff } (op = op' \Rightarrow arg = arg')$$

The only non-compatible combination of commands is two application of the setColor command, the idea being that you can not set the color twice in the same timestep.

We can now define the widget and prefix objects

**Definition 6.** *The widget object, denoted* Widget*, is indexed by $i \in \mathsf{Id}$ and is defined as*

$$\mathsf{Widget}_\infty\ i = \left\{(w, i) \mid w \in \mathcal{P}(\mathsf{Time} \times \mathsf{Cmd}), (t, c) \in w \wedge (t, c') \in w \to c \bowtie c'\right\}$$
$$\mathsf{Widget}_n\ i = \{(w, i) \subset \mathsf{Widget}_\infty\ i \mid \forall (t, c) \in w, t \leqslant n\}$$

*The prefix object, denoted* Prefix*, is indexed by $i \in \mathsf{Id}$ and $t \in \mathsf{Time}$ and is:*

$$\mathsf{Prefix}_\infty\ i\ t = \left\{(P, i) \subset \mathsf{Widget}_\infty\ i \mid \forall (t', c) \in P, t' \leqslant t\right\}$$
$$\mathsf{Prefix}_n\ i\ t = \begin{cases} \{(P, i) \subset \mathsf{Prefix}_\infty\ i\ t \mid \forall (t', c) \in P, t' \leqslant n\} & n < t \\ \mathsf{I} & \textit{otherwise} \end{cases}$$

The widget object is a collection of times and commands keeping track of what has happened to it at various times – imagine a *logbook* with entries for each time step. At the point at infinity, the "global" behavior of the widget is defined, i.e., the full logbook of the widget. For each $n$, $\mathsf{Widget}_n$ is simply what has happened to the widget so far, i.e., a truncated logbook. The prefix object is a widget object that is only defined up to some timestep, and is the unit after that. This yields a semantic difference between the widget where the color is set only once, and the widget where the color is set at every timestep. This reflects a real difference in actual widget behavior: if *turnRedOnClick w* later set to be blue, it will remain blue, but *keepTurningRed w* will turn back to being red.

To manipulate widgets we define two "restriction" maps.

**Definition 7.** *We define the following on widgets and prefixes*

$$\mathsf{shift}\ t : \mathsf{Widget}\ i \to_{\mathsf{Rel}_{\mathcal{R}}} \mathsf{Widget}\ i$$
$$(\mathsf{shift}\ t\ W)_n = \left\{(t' - t, c) \mid (t', c) \in W \wedge t \leqslant t'\right\}$$

$$\mathsf{prefix}\ t\ i : \mathsf{Widget}\ i \to_{\mathsf{Rel}_{\mathcal{R}}} \mathsf{Prefix}\ i\ t$$
$$(\mathsf{prefix}\ t\ i\ W)_n = \begin{cases} \{(t', c) \in W \mid t' < t\} & n < t \\ \mathsf{I} & n \geqslant t \end{cases}$$

The intuition behind these is that prefix $t\ i$ "cuts off" the widget after $t$, giving a prefix, whereas shift $t$ shifts forward all entries in the widget by $t$.

Using the above, we can now define the split and join morphisms. These are again given w.r.t ambient Id and Time objects, which will be part of the full hyperdoctrine structure:

**Definition 8.** *We define the following morphisms on the widget object*

$$\mathsf{split}\ i\ t : \mathsf{Widget}\ i \to_{\mathsf{Rel}_{\mathcal{R}}} \mathsf{Prefix}\ i\ t \otimes \mathsf{Widget}\ i\ @\ t$$
$$(\mathsf{split}\ i\ t\ w)_n = (\mathsf{prefix}\ t\ i\ w, \mathsf{shift}\ t\ w)_n$$

$$\mathsf{join}\ i\ t : \mathsf{Prefix}\ i\ t \otimes \mathsf{Widget}\ i\ @\ t \to_{\mathsf{Rel}_{\mathcal{R}}} \mathsf{Widget}\ i$$
$$(\mathsf{join}\ i\ t\ (p, w))_n = \begin{cases} p_n & n < t \\ w_{n-t} & n \geqslant t \end{cases}$$

***Linear-non-linear Hyperdoctrine*** So far we have not explained in details how to model the quantifiers in our system. To do this, we use the notion of a *hyperdoctrine* [22]. For first-order logic, this is a functor from a category of contexts and substitutions to the category of Cartesian closed categories, with the idea that we have one CCC for each valuation of the free first-order variables.

As our category of contexts, we use a Cartesian category to interpret our index objects, Time and Id. The former is interpreted as $\mathbb{N} + 1$ and the latter as $\mathbb{N}$. In our case, both Set and $\mathsf{Rel}_{\mathcal{R}}$ are themselves hyperdoctrines w.r.t to this category of contexts, the former a first-order hyperdoctrine and the latter a multiplicative intuitionistic linear logic (MILL) hyperdoctrine. Together these form a linear-non-linear hyperdoctrine through the adjunction given in Theorem 5.

**Definition 9.** *A linear-non-linear hyperdoctrine is a MILL hyperdoctrine $L$ together with a first-order hyperdoctrine $C$ and a fiber-wise monoidal adjunction $F : L \leftrightarrows C : G$.*

**Theorem 6.** *The categories* Set *and* $\mathsf{Rel}_{\mathcal{R}}$ *form a linear-non-linear hyperdoctrine w.r.t the interpretation of the indices objects, with the adjunction given as in Theorem 5.*

We refer the reader to the accompanying technical appendix for the full details.

***Denotational Semantics*** We the above, we have enough structure to give an interpretation of $\lambda_{\mathsf{Widget}}$. Again, most of this interpretation is standard in the use of the hyperdoctrine structure, and we interpret $\Diamond$ in the obvious way using the linear hyperdoctrine structure on $\mathsf{Rel}_{\mathcal{R}}$. As an example, we sketch the interpretation of the widget object and the setColor command below.

**Definition 10.** *We interpret the* Widget $i$ *and* Prefix $i$ *types using the widget and prefix objects:*

$$[\![\Theta \vdash \mathsf{Widget}\ i]\!] = \mathsf{Widget}\ [\![\Theta \vdash_s i : \mathsf{Id}]\!]$$
$$[\![\Theta \vdash \mathsf{Prefix}\ i\ t]\!] = \mathsf{Prefix}\ [\![\Theta \vdash_s i : \mathsf{Id}]\!]\ [\![\Theta \vdash_s t : \mathsf{Time}]\!]$$

*and we interpret the* setColor *commands as:*

$$[\![\mathsf{setColor} : \forall(i : \mathsf{Id}), \mathsf{Widget}\ i \otimes \mathsf{F}\ \mathsf{Color} \multimap \mathsf{Widget}\ i]\!] =$$
$$\{w \cup_W \{(0, (\mathsf{setColor}, col))\} \mid w \in [\![\mathsf{Widget}\ i]\!], col \in [\![\mathsf{Color}]\!]\}$$

*where $\cup_W$ is a "widget union", which is a union of sets such that identifiers indices and compatibility of commands are respected*

This interpretation shows that a widget is indeed a logbook of events. Using the setColor command simply adds an entry to the logbook of the widget. Note we only set the color in the current timestep. To set the color in the future, we combine the above with appropriate uses of splits and joins. The interpretation of split and join are done using their semantic counterparts, and the interpretation of onClick and onKeypress are done, using our non-deterministic semantics, by associating a widget with *all possible occurrences* of the corresponds event.

***Soundness of Substitution*** Finally, we prove that semantic substitution is sound w.r.t syntactic substitution. As with the proofs of type preservation for syntactic substitution, there are several cases for the different kinds of substitution, but the main results is again concerned with substitution of indices:

**Theorem 7.** *Given* $\zeta : \Theta' \to \Theta$, $\Theta; \Gamma \vdash_c e : X$ *and* $\Theta; \Gamma; \Delta \vdash_l t : A$ *then*

$$[\![\zeta]\!]\ [\![\Theta; \Gamma \vdash_c e : X]\!] = [\![\Theta'; \zeta(\Gamma) \vdash_c \zeta(e) : \zeta(X)]\!]$$
$$[\![\zeta]\!]\ [\![\Theta; \Gamma; \Delta \vdash_l t : A]\!] = [\![\Theta'; \zeta(\Gamma); \zeta(\Delta) \vdash_l \zeta(t) : \zeta(A)]\!]$$

Proofs for all six substitutions lemmas can be found in the technical appendix.

## Related and Future Work

Much work has aimed at a logical perspective on FRP via the Curry–Howard correspondence [21,18,17,19,20,10,1]. As mentioned earlier, most of this work has focused on calculi that have a Nakano-style later modality [25], but this has the consequence that it makes it easy to write programs which wake up on every clock tick. In this paper, we remove the explicit next-step modality from the calculus, which opens the door to a more efficient implementation style based on the so-called "push" (or event/notification-based) implementation style. Elliott [12] also looked at implementing a push-based model, but viewed it as an optimization rather than a first-class feature in its own right. In future work, we plan on implementing a language based upon this calculus, with the idea that we can compile to Javascript, and represent widgets with DOM nodes, and represent the $\Diamond A$ and $A \,@\, n$ temporal connectives using doubly-negated callback types (in Haskell notation, `Event A = (A -> IO ()) -> IO ()`). This should let us write GUI programs in functional style, while generating imperative, callback-based code in the same style that a handwritten GUI program would use.

Our model, in terms of $\mathsf{Set}^{\mathbb{N}+1}$, enriches LTL's semantics from time-indexed truth-values to time-indexed sets. The addition of the global view or point at infinity enables our model to distinguishes between least and greatest fixed points [15] (i.e., inductive and coinductive types), unlike in models of guarded recursion where guarded types are bilimit-compact [6]. This lets us encode temporal liveness and safety properties using inductive and coinductive types [10,2].

A recent development for comonadic modalities is the introduction of the so-called 'Fitch-style' calculi [7,11] as an alternative to the Pfenning–Davies pattern-style elimination [26]. These calculi have been used successfully for FRP [1], and one interesting question is whether they extend to adjoint calculi as well – i.e., can the $\mathsf{F}\ (X)$ modality support a direct-style eliminator?
